# A systematic review of the effectiveness and safety of Chinese herbal medicine formula Gualou Xiebai Banxia (GLXBBX) decoction for the treatment of stable angina pectoris

**DOI:** 10.1097/MD.0000000000018375

**Published:** 2019-12-20

**Authors:** Mingtai Chen, Ling Men, Haibin Wu, Guofu Zhong, Lijun Ou, Tao Li, Yingyi Guo, Haidan Lin, Jian Zhang, Dongcai Wang, Zhong Zhang

**Affiliations:** a Department of Cardiovascular Disease, Shenzhen Traditional Chinese Medicine Hospital, The Fourth Clinical Medical of Guangzhou University of Chinese Medicine; b Department of Nephrology, Shenzhen Traditional Chinese Medicine Hospital; c Health management department, Shenzhen Traditional Chinese Medicine Hospital, The Fourth Clinical Medical College of Guangzhou University of Chinese Medicine, Shenzhen, Guangdong Province; d Fuwai Hospital Chinese Academy of Medical Sciences, Beijing, China.

**Keywords:** GLXBBX decoction, Gualou Xiebai Banxia decoction, stable angina pectoris, systematic review

## Abstract

**Background:**

A growing number of studies suggest that Gualou Xiebai Banxia (GLXBBX) decoction, a well-known Chinese herbal formula, has beneficial effects on eliminating angina pectoris symptoms and improving condition of stable angina pectoris (SAP) patients. However, whether this treatment is effective and safe for SAP or not, evidence supporting the effectiveness and safety of this treatment is still incomplete. Besides, there is lack of systematic review to assess the detailed situation (including risk of bias and methodology) of current related clinical studies. This study aimed to evaluate the effectiveness and safety of GLXBBX in treating SAP.

**Methods:**

The major databases (MEDLINE, Embase, the Cochrane Library, China National Knowledge Infrastructure (CNKI), Chinese Scientific Journals Database (VIP) Database, Chinese Biomedical Database (CBM), Chinese Biomedical Literature Service System (SinoMed), and Wanfang Database) were searched from inception to March 2019. Randomized controlled trials (RCTs) of GLXBBX alone or combined with conventional drugs against conventional drugs for SAP were identified. Two assessors reviewed each trial independently. The methodological quality of the eligible studies was evaluated according to the Cochrane Collaboration's tool for assessing risk of bias. Both the data extraction and the literature quality screening evaluation were conducted independently by 2 researchers.

**Result:**

Totally 17 clinical RCTs were included in this study, involving 1676 patients. Due to the high probability of bias of the included studies, it was inappropriate to undertake a meta-analysis. Thus, we only conducted a systematic review and mainly discussed the methodology and limitation of the included studies.

**Conclusion:**

Although the current evidence prompted that GLXBBX might benefit SAP patients in improvement of angina pectoris, ECG, and blood lipid on a certain extent, this systematic review revealed no definite conclusion about the application of GLXBBX for SAP due to the poor methodological quality, high risk of bias, and inadequate reporting on clinical data. More rigorous, multicenter, sufficient-sample, and double-blind randomized clinical trials are warranted.

## 1 Introduction

Coronary heart disease (CHD) is known as one of the leading causes of morbidity and mortality worldwide.^[1]^ The major risk factors including dyslipidemia, diabetes mellitus, and hypertension contribute to the pathological process of CHD, which cause serious global economic burden.^[2–4]^ Stable angina pectoris(SAP), which refers to classic angina related to myocardial ischemia and is typically stimulated by activity, is one of the most common symptom of CHD.^[5]^ It has been estimated that more than half the patients with CHD were suffering from SAP.^[6]^ Not only can SAP affect patients’ daily activities, but also it can worsen their quality of life (QOL) regardless of physical health or mental health.^[7]^ The conventional treatment strategies, including revascularization, medication (anti-platelet therapy, anti-ischemic therapy, statins, and nitrates), and lifestyle modification, have been applied widely on stable angina patients, however, there still have been a large number of patients failing to relieve angina related symptoms completely and having varieties of adverse effects (including dizziness, headache, and nitrate-related tolerance).^[8–11]^ Due to disadvantages of conventional treatment strategies mentioned above, Chinese herbal medicine (CHM) may provide an alternative and additional therapy for SAP.

According to the CHM theory, SAP belongs to the CHM domain of “chest pain”, “heartache” majorly caused by “blood stasis”, “phlegm retention,” and “the deficiency in both Yang and Qi”.^[12–15]^ Gualou Xiebai Banxia (GLXBBX) decoction, which is a traditional Chinese herb medicine formula containing 3 major herbs (Gualou (*Trichosanthes kirilowii* Maxim.), Xiebai (*Allium macrostemon*), Banxia (*Rhizoma Pinelliae*)), has been used widely for treating chest pain in China since 25–220 AD.^[16]^ Currently, increasing evidence^[22–38]^ suggests that GLXBBX or modified GLXBBX decoction has beneficial effects on eliminating angina pectoris symptoms, and improving the electrocardiogram (ECG) for SAP patients.

### 1.1 Why it is important to do this review

Although the application of GLXBBX decoction is a commonly used ancient practice, there is still uncertainty about its effects. Moreover, the intensity of evidence has been poor and there has been lack of systematic analysis to assess the effectiveness and safety of GLXBBX for the treatment of SAP from randomized controlled trials. Therefore, this study aims to evaluate objectively the effectiveness and safety of GLXBBX decoction for SAP by integrating the existing trials.

## 2 Methods

### 2.1 Registration

The study protocol has been registered on international prospective register of systematic review (PROSPERO). The trial registration number of PROSPERO is CRD42018094538. The procedure of this protocol will be conducted according to the Preferred Reporting Item for Systematic Review and Meta-analysis Protocols (PRISMA-P) guidance.^[13]^

### 2.2 Eligibility criteria

#### 2.2.1 Types of studies

All the randomized controlled trials (RCTs) reporting the application of GLXBBX decoction for the treatment of SAP were included. Studies irrelevant to RCTs or trials that participants, control measures, interventions, and outcomes did not meet the criteria were excluded.

#### 2.2.2 Participants

All the participants enrolled in this study had to meet at least one of the current or past diagnostic criteria of SAP. The diagnostic criteria's included “Nomenclature and criteria for diagnosis of ischemic heart disease”^[17]^ or “ACC/AHA 2002 guideline update for the management of patients with chronic stable angina task force on practice guidelines (committee to update the 1999 guidelines)”^[18]^ or “Practice of internal medicine”.^[19]^

#### 2.2.3 Interventions

The experimental group was treated with modified GLXBBX alone or modified GLXBBX combined with conventional medicine treatment, and the same conventional medicine treatment must be used in the control group.

#### 2.2.4 Outcomes

The primary outcome measures will include: ① total effective rate of angina pectoris,^[20]^ clinical efficacy of angina pectoris is defined on 4 levels (significant improvement, improvement, no improvement, aggravation): significant improvement: the number of times of angina pectoris decreases by more than 80% or the consumption of nitroglycerin decreases by more than 80%; improvement: the number of times of angina pectoris decreases by 50% to 80% or the consumption of nitroglycerin decreases by 50% to 80%; no improvement: the number of times of angina pectoris and the consumption of nitroglycerin decreases by less than 50%; aggravation: the number of times, degree, and duration of angina pectoris increase or the consumption of nitroglycerin increases; the calculation formula: total effective rate of angina pectoris = (significant improvement cases + improvement cases)/total cases × 100%; ② total effective rate of electrocardiogram (ECG) improvement,^[20]^ clinical efficacy of electrocardiogram is defined on 4 levels (significant improvement, improvement, no improvement, aggravation): significant improvement: resting electrocardiogram returns to normal or double master's two-step test is negative or exercise tolerance increases by 2 levels compared with that before treatment in submaximal exercise test; improvement: ST-segment depression in resting electrocardiogram and double master's two-step test electrocardiogram recovers by more than 0.05 mV or the T-wave inversion in main leads recovers by more than 25% or the T-wave turns flat to upright or exercise tolerance increases by 1 levels compared with that before treatment in submaximal exercise test; no improvement: resting electrocardiogram or double master's two-step test electrocardiogram or submaximal exercise test electrocardiogram is almost the same as previously; aggravation: ST-segment depression in resting electrocardiogram and double master's two-step test electrocardiogram deepens by more than 0.05 mV or the T-wave inversion in main leads deepens by more than 25% or the vertical T wave becomes flat or the flat T-wave turns into inversion or exercise tolerance decreases by 1 levels compared with that before treatment in submaximal exercise test; the calculation formula: total effective rate of electrocardiogram improvement = (significant improvement cases + improvement cases)/total cases × 100%; The secondary outcome measures will include: levels of total cholesterol(TC), triglyceride (TG), low-density lipoprotein cholesterol (LDL-C), and high-density lipoprotein cholesterol (HDL-C) levels.

### 2.3 Search strategy

We searched electronic literature databases including MEDLINE, Embase, the Cochrane Library, China National Knowledge Infrastructure (CNKI), Chinese Scientific Journals Database (VIP) Database, Chinese Biomedical Database (CBM), Chinese Biomedical Literature Service System (SinoMed), and Wanfang Database. The retrieval time was from the inception date of the databases to March 2019. There was no limitation on the language of publication. Only clinical trials as a limitation were included and searched. Moreover, the search strategy for selecting the fields of title, abstract, or keyword was different referring to the characteristics of databases. Search terms were grouped into 3 blocks (see Table 1).

**Table 1:**
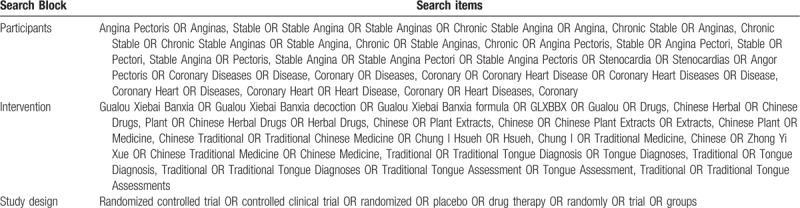
Search items.

### 2.4 Study selection and data extraction

Literature retrieved citations were managed by Note-Express software. Both the data extraction and the literature quality screening evaluation were conducted independently by 2 researchers (MC and ML). Studies which were duplicated or not accordant with eligibility criteria including types of studies, participants, interventions, and outcomes in this study were excluded. Disagreements were resolved by discussion or arbitrated by a third author (ZZ) if needed. The following data items were extracted: first author, publication year, sample size, gender, age, diagnosis standard, intervention and control measures, course of treatment, adverse effects report, and outcome assessment (see Table 2).

**Table 2:**
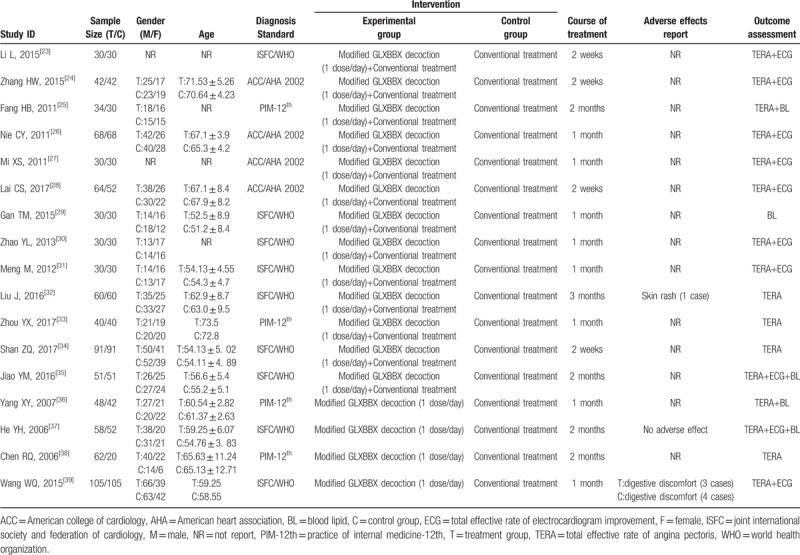
Basic characteristics of the included studies.

### 2.5 Risk of bias assessment

The methodological quality of the eligible studies was evaluated according to the Cochrane Collaboration's tool for assessing risk of bias.^[21]^ The assessment details included: sequence generation, allocation concealment, blinding of participants and personnel, blinding of outcome assessors, incomplete outcome data, selective reporting, and other sources of bias. Each assessment was graded into 3 levels: “low risk” or “high risk” or “unclear risk”. A funnel plot was applied to evaluating publication bias of the included studies. Funnel plots and Egger test were applied to evaluating publication bias of the included studies by Stata 12.0. Only qualitative evaluating risk of bias was conducted because included studies did not meet the criteria of meta-analysis in this review.

### 2.6 Data synthesis and statistical analysis

Statistical analyses were performed by RevMan 5.3 software provided by Cochrane Collaboration. Data was presented by risk ratio (RR) with its 95% confidence interval (CI) for dichotomous outcomes (total effective rate of angina pectoris and total effective rate of ECG improvement) and weighted mean difference (WMD) with its 95% CI for continuous outcomes (TC, TG, LDL and HDL). The *I*^2^ test was calculated to determine the amount of heterogeneity. According to the results of the heterogeneity test, a fixed effect model (*P* > .05, *I*^2^ < 50%) or a random effect model (*P* < .05, *I*^2^ > 50%) was selected. In this review, quantitative analysis could not be performed because included studies were only suitable to be qualitatively analyzed.

### 2.7 Sensitivity analysis and subgroup analysis

Sensitivity analysis or subgroup analysis was performed to analyse the heterogeneity or inconsistency among the studies and to explore potential sources of heterogeneity. We created a qualitative analysis when data extraction was insufficient.

### 2.8 Quality of evidence

The quality of evidence for each outcome was assessed according to the Grading of Recommendations, Assessment, Development, and Evaluation (GRADE) Handbook^[^^[22]^^]^ by 2 reviewers (MC and GZ) independently. Any disagreements about assessments were resolved by a third reviewer. The scores of GRADE were downgraded based on 5 factors:

1. Risk of bias (If there were poor trials with a high risk of bias and sensitivity analysis results had poor robustness through excluding the poor trials, evidence rated down by 1 level. If the most domains had unclear methodological bias risk, evidence rated down by 1 level),
2. Inconsistency (Inconsistency was assessed according to the outcomes of the χ^2^ test and *I*^2^ statistic reported in the systematic reviews. If *I*^2^ was >50%, *P* < .05, and the heterogeneity could not be explained by conducting subgroup analysis, the quality of evidence was downgraded),
3. Indirectness (If the participants, intervention, outcomes, or comparison had an indirect comparison in the study, evidence rated down by 1 level),
4. Imprecision (For dichotomous outcomes, if the number of events for each outcome was less than 300, evidence was downgraded. For continuous outcomes, if the number of events for each outcome was less than 400, evidence was downgraded),
5. Publication bias (Publication bias was assessed by presenting funnel plot and conducting Egger test. A two-tailed *P* value of <.05 was considered to indicate publication bias).

In this review, GRADE was unable to be performed because included studies were only suitable to be qualitatively analyzed.

### 2.9 Communicate with the authors of the primary studies

Two reviewers (MC and ZZ) contacted the authors of the included primary studies for detailed information if the information in the original article did not provide.

## 3 Results

### 3.1 Study identification

The process of study selection and identification is shown in PRISMA Flow Diagram. A total of 560 potentially relevant articles were initially screened in the electronic databases based on our literature searching strategy. After removing 222 duplicates, 338 articles were identified for further analysis. Through screening the titles and abstracts, 263 articles were excluded because they were not RCTs. A total of 75 full-text articles were retrieved for further assessment, of which 58 were excluded for the following reasons: improper participants (n = 17); irrelevant intervention measures (n = 7); irrelevant control measures (n = 15); inappropriate types of studies (n = 8); inappropriate outcome measures (n = 11). Finally, 17 full-text articles were then assessed for eligibility.

### 3.2 Study characteristics

Essential characteristics of 17 studies^[23–39]^ including 1676 cases are shown in Table 2. 17 studies including 873 patients from experimental treatment group and 803 patients from conventional treatment group were enrolled into this systematic review. All of these trials were carried out in China and all studies were of small sample size, ranging from 60 to 210 participants. The average age of patients enrolled in the studies ranged from 51 to 73 years of age. Three diagnostic criteria of SAP were specified: 9 trials^[23,29–32,34,35,37,39]^ used “Nomenclature and criteria for diagnosis of ischemic heart disease”; 4 trials^[24,26–28]^ used “ACC/AHA 2002 guideline update for the management of patients with chronic stable angina task force on practice guidelines (committee to update the 1999 guidelines)”; 4 trials^[25,33,36,38]^ used “Practice of internal medicine”. All the studies used a two-arm design (1 experimental group vs 1 conventional treatment group). For interventions, patients in the experimental group received either GLXBBX alone (n = 4)^[36–39]^ or GLXBBX combined with conventional treatment (n = 13).^[23–35]^ Patients in the conventional treatment group were treated with conventional treatment alone including antiplatelet therapy, anti-ischemic therapy, statins, and nitrates. The primary outcomes of total effective rate of angina pectoris were reported in 16 studies^[23–28,30–39]^ and the total effective rate of ECG improvement outcomes were reported in 10 studies.^[23,24,26–28,30,31,35,37,39]^ In addition, the blood lipid level outcomes of TC, TG, LDL, and HDL were reported in 5 studies.^[25,29,35–37]^ The treatment duration of the studies ranged from 2 weeks to 12 weeks.

### 3.3 Risk of bias within studies

**Figure 1:**
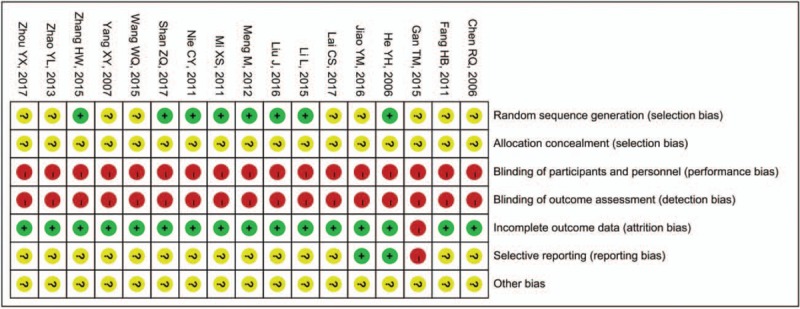
Risk of bias summary.

**Figure 2:**
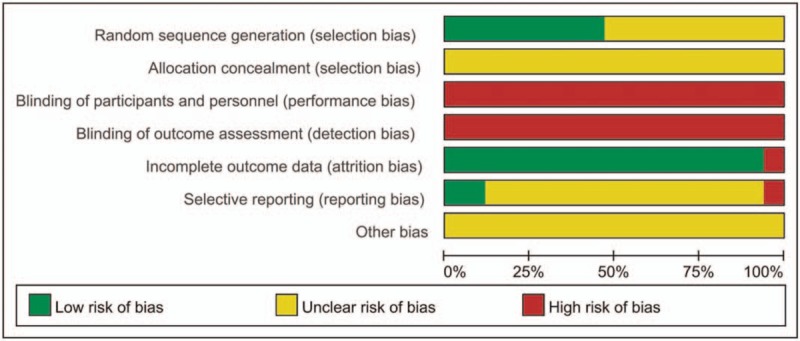
Risk of bias graph.

The methodological quality of the included studies is described in Figures 1 and 2. Although randomization was declared among the trials, only 8 studies^[23,24,26,27,31,32,34,35]^ stated the method of the sequence generation with random number table and drawing, while none of the 17 studies reported details for sample size calculations and intentional treatment analysis. All trials did not mention the random allocation concealment, indicating the bias of selection were unclear. Moreover, none of trials was double-blind and there was no binding of outcome assessment in all trials, showing high risk of performance bias and detection bias. All studies except 1 trial^[29]^ lacking of total effective rate of angina pectoris showed a low risk of bias for incomplete outcome data. Furthermore, 2 trials^[35,37]^ were evaluated as low risk of reporting bias because of their complete outcome measures, while 15 trials were evaluated as unclear^[23–28,30–34,36,38,39]^ or high risk^[29]^ of reporting bias due to their insufficient outcome measures. Last but not least, the risk of other bias of all trials was assessed as unclear because no protocols or other information could be obtained from the primary authors.

### 3.4 Data synthesis and statistical analysis

In this review, it was implied that all of trials declared as randomized trials in Chinese databases might not be real RCTs due to methodological shortcomings and publication bias. In addition, almost all of the included trials had drawbacks in the methodological design, including generation of randomization, concealment of allocation, and inadequate reporting on blinding, dropouts, and the pre-estimation of sample size. Therefore, the data in all studies was inadequate to be used in quantitative synthesis (meta-analysis). However, we still conducted a qualitative synthesis and systematic review, mainly discussed the issue on the RCT methodology and limitations of included studies.

### 3.5 Outcome measures

#### 3.5.1 Total effective rate of angina pectoris

16 trials,^[23–28,30–39]^ including 843 patients in the experimental group treated with GLXBBX decoction alone or GLXBBX decoction combined with control group treatment and 773 patients in the control group treated with conventional treatment, reported the total effective rate of angina pectoris for SAP. All of the trials reported that the total effective rate of angina pectoris in either GLXBBX decoction alone or GLXBBX decoction combined with control group treatment appeared superior to that in control group treatment. However, there were some concerns that we had to consider. Firstly, not all of studies provided the detailed information about how to record the patient's situation when the angina pectoris occurred in the article. Therefore, we tried to contact the authors of included studies and they responded that SAP patients’ number of times of angina pectoris and the consumption of nitroglycerin were recorded every week and the observations were stationary in all studies. In addition, all of the studies did not provide the detailed information about accurate recorded data and average frequency of angina pectoris even if we asked for that.

#### 3.5.2 Total effective rate of ECG improvement

A total of 10 trials^[23,24,26–28,30,31,35,37,39]^ reported the total effective rate of ECG improvement for SAP. There were 948 patients in total, including 480 patients in the experimental group treated with GLXBBX decoction alone or GLXBBX decoction plus control group treatment and 468 patients in the control group treated with conventional treatment. All of the trials reported that the total effective rate of ECG improvement in either GLXBBX decoction alone or GLXBBX decoction combined with control group treatment appeared superior to that in control group treatment. Nevertheless, there were also some doubts that we should consider. Firstly, even all of the trials reported that they recorded the ECG before and after treatment, but the detailed information about how to record and evaluate the patient's ECG was not provided. Additionally, no trial mentioned that the situation when patients’ angina pectoris occurred but the ECG did not change or ECG could not reflect the course of stenocardia in the articles. Moreover, no trial reported that how to recroded when ECG could not reflect the state of overt angina patients but submaximal exercise test could not be used in patients with overt angina. Finally, we have asked authors for detailed information but we have not received definite and satisfactory answers.

#### 3.5.3 Blood lipid level improvement

A total of 5 trials^[25,29,35–37]^ reported the improvement of blood lipid levels (TC, TG, LDL, HDL). There were overall 426 patients, including 221 patients in the experimental group and 205 patients in the control group. All of the trials showed that the TC level in the experimental group was significantly lower than that in the control group. Only 3 trials^[25,35,37]^ reported that the TG, LDL, and HDL level in the experimental group were significantly better than that in the control group, when there was no significant difference between TG, LDL, and HDL level in the experimental group and the control group. However, all the trials’ sample size were small and the total sample size of 5 trials was not large enough. Therefore, the results above might be inconclusive and needed further investigation.

### 3.6 Adverse effects

Adverse effects were reported in 2 trials^[32,39]^ and not mentioned in others. Onetrial^[32]^ reported that there was 1 patient with skin rash in control group, while another trial^[39]^ observed that there were 3 cases of digestive system symptoms (including nausea, dyspepsia, diarrhea, and constipation) in experimental group and 4 cases of digestive system symptoms in control group. However, none of the adverse effects were serious and patients recovered soon without additional treatment.

### 3.7 Sensitivity analysis and subgroup analysis

The extraction data was insufficient and inadequate, therefore we only created a qualitative analysis.

### 3.8 Publication bias

Due to insufficient details of the outcomes, funnel plot to evaluate publication bias were not conducted. Therefore, only qualitative evaluation of publication bias could be carried out. According to the limitations of studies, the overall quality of most studies were not high. Moreover, the results of total effective rate of angina pectoris and total effective rate of ECG improvement in most studies were positive, so 1 possibility that positive results increased the success rate of publication could not be excluded. Consequently, publication bias in this study was not clear and objective conclusion could only be made after further evaluation methods of publication bias such as funnel plot qualitative evaluation and Egger, Begg quantitative evaluation.

### 3.9 Quality of evidence

In this review, GRADE was unable to be performed because included studies were only suitable to be qualitatively analyzed.

### 3.10 Patient and public involvement

Patients and or public will not involved due to this study belonging to the secondary sources analysis.

### 3.11 Discussion summary of therapy effectiveness

Coronary heart disease (CHD) is still the leading cause of mortality and disability in the world, the burden of which has been estimated to rise to 82 million disability-adjusted life years (DALYs) globally in 2020.^[40–43]^

Stable angina pectoris (SAP) is one of the most common symptom of CHD.^[44–46]^ Although most of SAP patients have been on the conventional western treatment, the treatment situation of which has not been completely satisfactory yet due to the patients’ persistent discomfort and varieties of drug adverse effects.^[3,47]^ As a result, it is of great importance to explore more effective and safer prevention and treatment. In China, Chinese herbal medicine (CHM), such as GLXBBX decoction having a long history of integration with conventional medical interventions, has been widely applied on SAP.^[44,48–51]^ However, the effectiveness and safety has been controversial worldwide because of the lack of systematic review summarizing the existing evidence. Thus, this manuscript aims to provide objective evidence by systematically analysing the effectiveness and safety of GLXBBX decoction on SAP from aspects of total effective rate of angina pectoris, ECG improvement, blood lipid improvement, and adverse effects. Seventeen claimed trials, with a total of 1676 patients with SAP, met the inclusion criteria and were included in this review. First of all, the results of 16 trials^[23–28,30–39]^ showed that the GLXBBX decoction combined with conventional treatment or GLXBBX decoction alone was associated with better outcomes of total effective rate of angina pectoris. Moreover, the results of 10 trials^[23–24,26–28,30–31,35,37,39]^ also suggested that the GLXBBX decoction combined with conventional treatment or GLXBBX decoction alone treatment was associated with superior outcomes of ECG improvement to conventional treatment. In addition, 5 trials,^[25,29,35–37]^ which were screened to systematically analyze the blood lipid improvement, involving 426 SAP patients. All of the 5 trials showed that the TC level in the experimental group was superior to that in the control group. Only 3 trials^[25,35,37]^ reported that the TG, LDL, and HDL level in the experimental group were significantly better than that in the control group.

Results above suggested that GLXBBX treatment tended to be of benefit to reduction of angina symptom and ECG improvement, besides, the effectiveness of GLXBBX treatment to improve blood lipid level in SAP patients was uncertain. However, the methodological quality of the trials was evaluated generally as low and there were few (only 5) trials reporting blood lipid improvement, in which a single trial result accounted for a large proportion, causing large heterogeneity and unstable results. Therefore, the conclusion should be interpreted cautiously and confirmed by further study.

### 3.12 Summary of therapy safety

There was incomplete reporting on ADEs/ADRs in the included trials. The number of suspected adverse events were reported in both treatment group and control group (3/5). The main adverse reactions above included digestive system symptoms and skin rash, which could be tolerated by patients and recovered soon without additional treatment. Nevertheless, none of precise and reliable quantitative estimations of relationship likelihood about these ADEs/ADRs were provided in current review. In addition to this, the adverse reactions could be caused by GLXBBX or combined drugs, such as aspirin, and the diagnosis of ADRs might differ from different trials according to trials’ design, the trials’ quality and so forth. Further studies to explore the causality assessment on these ADRs were needed. Furthermore, it was of difficulty to record ADRs because of the inadequate reporting and short course of treatment. Consequently, further research defining the ADRs of GLXBBX and investigating factors that might cause ADRs of GLXBBX in long term use of GLXBBX was needed.

### 3.13 Limitations

Above all, methodological quality of most included trials was limited and the specific problems were as follows. All the included studies had several unclear risks of bias according to the Cochrane Collaboration’ Risk of bias’ s tool. All trials claimed randomization, however, only 8 trials^[23–24,26–27,31–32,34,37]^ reported the method of the sequence generation with random number table or drawing, and the other 9 trials^[25,28–30,33,35–36,38–39]^ only mentioned “randomly allocating” without details. Besides, all the trials did not describe allocation concealment in detail and blinding of participants and personnel were unclear, which led to a difficulty to judge whether the trials were conducted appropriately. There was no trial reporting estimation of sample size and no multi-center, large-scale trial identified, which gave rise to reducing statistical power to identify the therapeutic effect. In addition, most of trials were evaluated as unclear^[23–28,30–34,36,38–39]^ or high risk^[29]^ of reporting bias due to their insufficient outcome measures. Moreover, none of the trial mentioned dropouts, withdrawals, and follow-up visit which reported rate of angina recrudescence, response to therapy, adverse effects and so on. In addition, more detailed patient characterization and sampling representativeness are needed. However, we have not got reply yet after we contacted the authors of included trials.

Secondly, heterogeneity was another vital issue that should be taken into consideration. There was significant heterogeneity in TC, TG, LDL, and HDL level analyses of 5 trials^[25,29,35–37]^ which compared GLXBBX plus conventional treatment or GLXBBX alone treatment with conventional treatment. One of the possible reason for presence of heterogeneity might be the treatment duration of the trials, which could be inferred from the results of subgroup analysis according to the course of treatment. However, because of the insufficient number of the trials for meaningful and accurate subgroup analysis, the results might be unstable and needed further investigation. Another probable reason for that might be the various different diagnosis criteria in different trials. Moreover, the methodological quality of the trials^[25,29,35,36]^ included was limited, only 1 trial^[37]^ mentioning random sequence generation and with less biases was scored as superior to the other trials. Furthermore, although the wide application of GLXBBX treatment on SAP patients in China indicated the effectiveness, there has not been any multi-center, large scale trial found to provide convincing evidence. Those small sample size trials with positive results were reported in most of included studies and some other negative results might not be reported. Besides, as the definition of “total effective rate of angina pectoris” and “total effective rate of electrocardiogram improvement” mentioned above, the alleviating angina pectotis effect of the interventions depended on the number of times of angina pectoris and the consumption of nitroglycerin, the changes of resting electrocardiogram or electrocardiogram of treadmill exercise test were compared before and after the intervention. However, the more detailed information of the method to record the data and specific parameters were not reported. Therefore, it would be more accurate, convincing and objective for the included studies if the detailed information about the number of times of angina pectoris, the consumption of nitroglycerin, resting electrocardiogram and electrocardiogram of treadmill exercise test was provided. In addition, total effective rate of angina pectoris, one of the primary outcome measures, was suitable for patients in stationary stage. However, surrogate outcome measures such as total effective rate of electrocardiogram improvement and cholesterol were inappropriate and insufficient to evaluate the effectiveness of GLXBBX treatment on SAP patients. Instead, major adverse cardiovascular events such as unstable angina, myocardial infarction, and sudden death should be included to evaluate the effectiveness of GLXBBX treatment. Consequently, qualified study should include a long-term, rather than a short-term (less than 3 months), outpatient follow-up process to record the major adverse cardiovascular events. Last but not least, the publication bias of the studies could not be excluded and more evaluation of publication bias such as funnel plot qualitative evaluation and Egger, Begg quantitative evaluation should be taken into account.

## 4 Conclusion

From this systematic review, there was insufficient evidence to revealed that comparing with conventional treatment as monotherapy, GLXBBX combined with conventional treatment or GLXBBX alone treatment could significantly improve total the effective rate of angina pectoris, ECG and the blood lipid level of SAP patients. Consequently, the results should be interpreted with caution owing to the poor methodological quality, some possible risks of bias and inadequate reports on clinical data. Therefore, in order to definite the effectiveness and safety of GLXBBX treating SAP, more rigorous, larger scaled, and multi-center randomized clinical trials with long-term follow-up are warranted for convincing evidence in the future.

## 5 Ethics and dissemination

This review does not require the ethical approval because there are no concerns about the patients’ privacy. The results of the meta-analysis will be reported according to the PRISMA extension statement and disseminated in a peer-reviewed journal.

## Author contributions

**Conceptualization:** Mingtai Chen, Haibin Wu, Dongcai Wang, Jian Zhang, Zhong Zhang.

**Data curation:** Mingtai Chen.

**Formal analysis:** Ling Men, Tao Li.

**Funding acquisition:** Haibin Wu, Dongcai Wang, Jian Zhang, Zhong Zhang.

**Investigation:** Ling Men, Jian Zhang.

**Methodology:** Tao Li, Haidan Lin.

**Project administration:** Guofu Zhong, Zhong Zhang.

**Resources:** Guofu Zhong.

**Software:** Guofu Zhong, Haidan Lin.

**Supervision:** Yingyi Guo.

**Validation:** Lijun Ou, Yingyi Guo, Haidan Lin.

**Visualization:** Mingtai Chen, Lijun Ou.

**Writing – original draft:** Mingtai Chen, Ling Men.

**Writing – review & editing:** Mingtai Chen, Ling Men.

Mingtai Chen orcid: 0000-0003-4579-5559.
